# The Crohn’s disease associated SNP rs6651252 impacts *MYC* gene expression in human colonic epithelial cells

**DOI:** 10.1371/journal.pone.0212850

**Published:** 2019-02-22

**Authors:** Stephen M. Matthews, Melanie A. Eshelman, Arthur S. Berg, Walter A. Koltun, Gregory S. Yochum

**Affiliations:** 1 Department of Biochemistry & Molecular Biology, Pennsylvania State University College of Medicine, Hershey, Pennsylvania, United States of America; 2 Department of Surgery, Division of Colon and Rectal Surgery, Pennsylvania State University College of Medicine, Hershey, Pennsylvania, United States of America; 3 Department of Public Health Sciences, Pennsylvania State University College of Medicine, Hershey, Pennsylvania, United States of America; Hirosaki University Graduate School of Medicine, JAPAN

## Abstract

Crohn’s disease (CD) is a debilitating inflammatory bowel disease (IBD) that arises from chronic inflammation in the gastrointestinal tract. Genome-wide association studies (GWAS) have identified over 200 single nucleotide polymorphisms (SNPs) that are associated with a predisposition for developing IBD. For the majority, the causal variant and target genes affected are unknown. Here, we investigated the CD-associated SNP rs6651252 that maps to a gene desert region on chromosome 8. We demonstrate that rs6651252 resides within a Wnt responsive DNA enhancer element (WRE) and that the disease associated allele augments binding of the TCF7L2 transcription factor to this region. Using CRISPR/Cas9 directed gene editing and epigenetic modulation, we find that the rs6651252 enhancer regulates expression of the *c-MYC* proto-oncogene (*MYC*). Furthermore, we found *MYC* transcript levels are elevated in patient-derived colonic segments harboring the disease-associated allele in comparison to those containing the ancestral allele. These results suggest that Wnt/MYC signaling contributes to CD pathogenesis and that patients harboring the disease-associated allele may benefit from therapies that target MYC or MYC-regulated genes.

## Introduction

Crohn’s disease (CD) and ulcerative colitis (UC) are the two main classes of inflammatory bowel disease (IBD), and arise from chronic inflammation in the gastrointestinal (GI) tract [1]. CD can present anywhere along the GI tract whereas UC is confined primarily to the colon [1,2]. In a generally accepted view, IBD results from one or more environmental triggers in a genetically susceptible individual [3,4]. While the precise environmental exposure is debatable, that fact that 5–23% of IBD patients have a first-degree relative that is also afflicted with disease is supportive of a genetic inheritance [5].

Numerous genome-wide association studies (GWAS) have been conducted to identify genetic variants associated with IBD predisposition [3]. In a landmark study, Jostins et al. reported results from a meta-analysis of GWAS data generated from sporadic and familial IBD patients [6]. Altogether, 163 single nucleotide polymorphisms (SNPs) that conferred IBD susceptibility were identified, of which 110 were associated with both CD and UC [6]. Of the remaining 53, 30 were specific for CD and 23 were specific for UC [6]. Several disease variants produced missense mutations within the nucleotide oligomerization domain two (NOD2) protein that senses bacterial peptidoglycans, and the autophagy protein ATG16L1. Other variants were also identified in the IL23 receptor and the HLA locus, and together, these findings substantiate the notion that IBD manifests, in part, through a deregulated immune response to commensal or pathogenic bacteria in the gut lumen [3,6]. Since that time, the list of variants associated with IBD has grown to include over 200 distinct loci [2,6–8]. While these results clearly demonstrate the power of GWAS to inform on disease pathogenesis, approximately 80–90% of the identified alleles map to non-coding regions of the genome, many of which are in gene-poor regions [6]. How these non-coding variants are associated with IBD is largely unknown, and this remains a significant obstacle in our understanding of the genetic basis for disease pathogenesis.

Studies from the encyclopedia of DNA elements (ENCODE) consortium [9,10], and from others [11,12], indicate the majority of common disease-associated variants map to gene regulatory regions of the genome, also known as enhancer elements. Indeed, Mokry et al. found that 27% of the 163 IBD associated SNPs directly overlapped a putative enhancer element, as defined by regions containing elevated levels of histone H3 that is acetylated on lysine 27 (H3K27Ac) [13]. Consideration of regions in linkage disequilibrium increased the total number of SNPs associated with active enhancer elements to 56% of the 163 SNPs [13]. While several IBD-associated SNPs were confirmed to demarcate enhancer elements, the precise impact of these variants on enhancer function, the upstream signaling pathways involved and the relevant downstream transcription factors have not been adequately addressed.

The intestines are lined by a single layer of epithelial cells that protect the underlying mucosa and sub-mucosa from toxic contents of the gut lumen and the microbiota [14]. Because epithelial cells are subjected to damage, the entire epithelial layer is replaced every five to six days making the intestines one of the most highly regenerative organs in the body [14,15]. The Wnt/β-catenin signaling pathway contributes to this regenerative process by driving cellular proliferation [16]. In the presence of Wnt, the β-catenin transcriptional co-activator translocates to the nucleus and associates with members of the T-cell factor/ lymphoid enhancer factor (TCF/LEF; hereafter, TCF) family of transcription factors [17,18]. TCF7L2 is the predominant TCF family member expressed in intestinal epithelial cell lines [19,20]. β-Catenin/TCF complexes bind to Wnt-responsive DNA regulatory elements (WREs) and primarily increase expression of target genes [18]. One critical target in the intestines is the *c-MYC* (*MYC*) proto-oncogene [21–23]. MYC is a transcription factor that promotes cellular growth and proliferation through increasing expression of genes involved in the cell cycle, ribosome biogenesis and metabolism [24,25]. While several WREs localize to the proximal promoter of the *MYC* gene, many also map tens to hundreds of kilobases away and are juxtaposed to *MYC* through long-range chromatin loops [26].

In this study, we investigate the CD-associated SNP, rs6651252, which maps to a gene-poor region on chromosome 8 [6]. We demonstrate that this SNP impacts a novel WRE that controls *MYC* expression in intestinal epithelial cells, and that patient intestinal tissues harboring the disease-associated allele display increased levels of *MYC* transcripts. These findings offer one explanation for how Wnt/MYC signaling may contribute to CD pathogenesis and raise the possibility that patients harboring this allele may benefit from MYC targeted therapies.

## Materials and methods

### Cell lines

The HCT116 and DLD-1 cell lines were obtained from the American Type Culture Collection (cat. numbers CCL-221 and CCL-247) while HEK293T cells were obtained from Invitrogen. HCT116 and HEK293T cells were maintained in DMEM (Corning) supplemented with 10% FBS, 5 mM L-glutamine, and 1% penicillin/streptomycin. DLD-1 cells were maintained in RPMI supplemented with 10% FBS and 1% penicillin/streptomycin. Cells were cultured in an incubator at 37°C in 5% CO_2_.

### Plasmids

To generate the rs6651252 WRE luciferase plasmid (rs6651252-luc), a 555 bp DNA segment containing rs6651252 was amplified by PCR with genomic DNA isolated from HCT116 cells using the DNAEasy kit (Qiagen) serving as the template. The PCR product was subcloned into the pGL3-promoter luciferase vector (Promega) as a KpnI-NheI fragment. Site-directed mutagenesis was conducted to convert the ancestral T variant to the disease-associated C variant using the QuickChange mutagenesis kit (Agilent) following the manufacturer’s instructions. To generate rs6651252 WRE expanded luciferase construct [rs6651252 (exp)-luc], a 2.7 kb segment containing the rs6651252 WRE was amplified by PCR and the product was likewise subcloned as a KpnI-NheI fragment into the pGL3-promoter luciferase vector. Primer sequences used in the PCR reactions can be found in S1 Table.

For the epigenetic silencing assay, the phU6-sgRNA plasmid and the pLV hU6-sgRNA hUbC-dCas9-KRAB-T2a-Puro plasmid encoding dCas9-KRAB fusion protein was obtained from Addgene (#53188 and #71236, respectively). Using the 2.7 kb segment of DNA, the CRISPR guide RNA design tool (crispr.mit.edu) was used to identify unique guide sequences. For each of eight guides, paired oligonucleotides were designed with BbsI overhanging restriction sites to facilitate subcloning into the phU6-gRNA plasmid vector. Complementary DNA oligonucleotides (10 μM) corresponding to each of the eight guide RNA sequences, were annealed in 1x T4 ligase buffer by heating to 95°C for 5 min and cooling 5°C/min to room temperature. To digest and anneal sgRNA, 25 ng of pU6-gRNA plasmid was mixed with 1 μl annealed guide pair, 1 μl of 10x T4 ligase buffer, 2.5U BbsI, 0.5 μl of T4 ligase and 6 μl H_2_O. The reactions were subjected to 25 cycles at 37°C for 5 min and 23°C for 5 min on a DNAengine thermocycler (BioRad). Primer sequences used to generate the guide RNAs can be found in S1 Table.

To create the rs6651252 WRE deletion cell line, guide RNAs were designed to flank a 692 bp region surrounding rs6651252, and corresponding oligonucleotides were annealed as described above. The fragments were ligated into the pSpCas9(BB)-2A-GFP (PX458) CRISPR/Cas9 plasmid (Addgene, #48138), which was first digested with BbsI. Primer sequences used to generate guide RNAs and to assess the rs6651252 status are listed in S1 Table. Sanger sequencing was used to verify each plasmid insert and enhancer deletions in the knockout clones.

### Chromatin Immunoprecipitation (ChIP)

ChIP assays were performed as previously described [27], using approximately 6.0 x 10^6^ cells per sample. The cross-linked and sheared DNA was precipitated with 3 μg of the following antibodies; TCF4 (05–511 Millipore), β-catenin (610154, BD scientific), H3K27Ac (ab4729, Abcam) and FLAG (F1804-200UG, Sigma). Precipitated and purified DNA was analyzed through qPCR with primers listed in S1 Table. Reactions were run on a MyIQ real-time PCR machine (Biorad) using cycling parameters previously described [27].

### Luciferase reporter assays

Luciferase assays were conducted as described previously [22]. Briefly, approximately 2.5 x 10^4^ cells were seeded per well in a 24-well plate. Transfections were conducted using Lipofectamine 2000 following manufacturer’s guidelines. Each reaction contained 50 ng of the luciferase reporter plasmid, and 2 ng pLRL-SV40 Renilla, which served as a transfection control. Where indicated, 50 ng of pcDNA3.1-β-catenin S45F [28], 50 ng of pME18 Lef [28], and 50 ng of pLV hU6-sgRNA hUbC-dCas9-KRAB-T2a-Puro were added to the transfection. The phU6-sgRNA plasmids encoding the guide RNAs (25 ng each) were included as indicated. Total concentration of DNA was adjusted to 2 μg per reaction using pBluescript (Stratagene). Transfection mixtures were incubated on cells for 6 h, after which the media was replaced with normal growth media. Each reaction was conducted in quadruplicate. After 24 h, cells were lysed in 200 μl passive lysis buffer and luciferase levels were measured using the dual luciferase assay kit (30005–2; Biotium) on a Glomax 20/20 single chamber luminometer (Promega).

### CRISPR/Cas9-mediated deletion of the rs6651252 WRE

CRISPR/Cas9 modified clonal HCT116 cell lines were generated following the protocol outlined previously [29]. Briefly, 500 ng of each CRISPR/Cas9 plasmid (PX458), encoding guide RNA sequences that flanked rs6651252, were transfected into HCT116 cells using lipofectamine 2000 (Invitrogen) for 6 h. After 24 h, the cells were harvested and a FACSDiva (Becton Dickinson) machine was used to seed 2 cells per each well of a 96-well plate. After expanding the clones, genomic DNA was isolated using the Lyse&Go kit (Pierce) and the rs6651252 region was amplified by PCR using the DreamTaq Green (Thermo) polymerase and primers listed in S1 Table. The products were resolved on a 1% agarose gel, excised with a scalpel, and purified using the MinElute PCR Purification Kit (Qiagen). The products were sequenced to identify clones harboring rs6651252 WRE deletions.

### DNA pull-down assay

The DNA pull-down assay was conducted as previously described with minor modification [30]. Probes were designed with 10 nucleotides flanking each side of the TCF consensus motif within the rs6651252 WRE. The paired probes were annealed as described previously [30]. The annealed probes (15 μM) were incubated with 0.1 mg of streptavidin coated magnetic beads (Promega Z5481) for 1.5 h at room temperature. Prior to use, the beads were washed three times in 1X sodium citrate buffer (30 mM NaCl, 0.035 mM C_6_H_5_O_7_Na_3_) and then resuspended in 100 μl binding buffer (1M NaCl, 10 mM Tris, 1 mM EDTA, pH = 8.0). To prepare the protein lysates, 2.5 x 10^7^ HCT116 cells were harvested and lysed in RIPA buffer supplemented with freshly added protease inhibitors (1 mM PMSF, 10 μl/ml aprotinin, 10 μg/ml leupeptin). For each binding reaction, 200 μg of protein lysate was incubated at 4°C with 150 μg sonicated salmon sperm for 30 min to reduce non-specific interactions. This lysate was added to probes conjugated with the magnetic beads and the reactions were incubated for 2 h at room temperature on a rotating platform. The protein/DNA complexes were collected using a magnetic stand, washed three times in RIPA buffer, and eluted in 50 μl of 2x laemmli loading buffer. The proteins were resolved on an 8% polyacrylamide gel and standard western blot analysis as previously described [31]. The blots were incubated overnight with anti-TCF7L2 antibodies (05–511 Millipore, 1:750 dilution) followed by incubation with HRP conjugated anti-mouse secondary (Jackson Immunoresearch, 1:5000 dilution) for 2 h prior to ECL treatment and exposure to film. In competition assays, 250 ng, 500 ng, or 1500 ng of annealed and unlabeled probes were added concurrently to the binding reactions containing the indicated biotinylated probes. Probe sequences are listed in S1 Table.

### CRISPR/dCas9 repression assay

In the epigenetic repression assays, 250 ng of the pLV hU6-sgRNA hUbC-dCas9-KRAB-T2a-Puro plasmid and 25 ng each of the phU6-gRNA plasmids harboring guide RNAs that tiled the 2.7 kb segment containing the rs6651252 WRE were electroporated into 1.0 x 10^7^ HCT116 or HEK293T cells using the Amaxa Cell line Nucleofector Kit V (Lonza) and an Amaxa Nucleofector II electroporator following the manufacturers guidelines. *MYC* gene expression was assessed by RT-qPCR 72 h after electroporation.

### Quantitative reverse transcription PCR

To assess *MYC* gene expression, RNAs were collected and cDNAs synthesized using protocols that were described previously [29]. For experiments involving patient tissues, approximately 1 g of flash-frozen and full thickness colonic tissue was homogenized in 1 ml of TRIzol (Thermo Fisher) in an eppendorf tube using a disposable micropestle [32]. Following a 5 min incubation at room temperature, 200 μl of chloroform was added and the mixture shaken vigorously before centrifugation at 12,000 x *g* for 15 min at 4°C. The upper layer was removed, mixed with an equal volume of 100% ethanol, and the RNAs were purified using an RNeasy Mini kit (Qiagen). For both cell line and patient sample experiments, cDNA was synthesized from 500 ng of RNA using the iScript cDNA synthesis kit (BioRad), following manufacturer protocol. Data are presented as relative levels using the 2^ΔCT^ method with *GAPDH* and *TUBB3* serving as the reference genes. Primer sequences used in the RT-qPCR experiments are listed in S1 Table.

### Patient samples

The samples evaluated in this study were collected with patient consent as part of the biorepository within the Department of Surgery, Division of Colon and Rectal Surgery at the Pennsylvania State University College of Medicine. The Pennsylvania State University College of Medicine Institutional Review Board (IRB) evaluated and approved protocol number HY98-057EP-A “A Proposal for Creation of an Inflammatory Bowel Disease Registry” for creation and maintenance of our biorepository. The allelic status of IBD associated SNPs is assessed using a custom oligonucleotide array [33].

### Statistics

Each experiment was performed at least three times. For ChIP-qPCR and RT-qPCR, each sample was amplified in quadruplicate reactions per experiment. Significance of the results was evaluated with either a pairwise Student’s T-test or one-way ANOVA with a Tukey test. Data were considered significant for p-value less than 0.05.

## Results

### β-Catenin and TCF7L2 bind the rs6651252 locus in colonic epithelial cells

**Fig 1:**
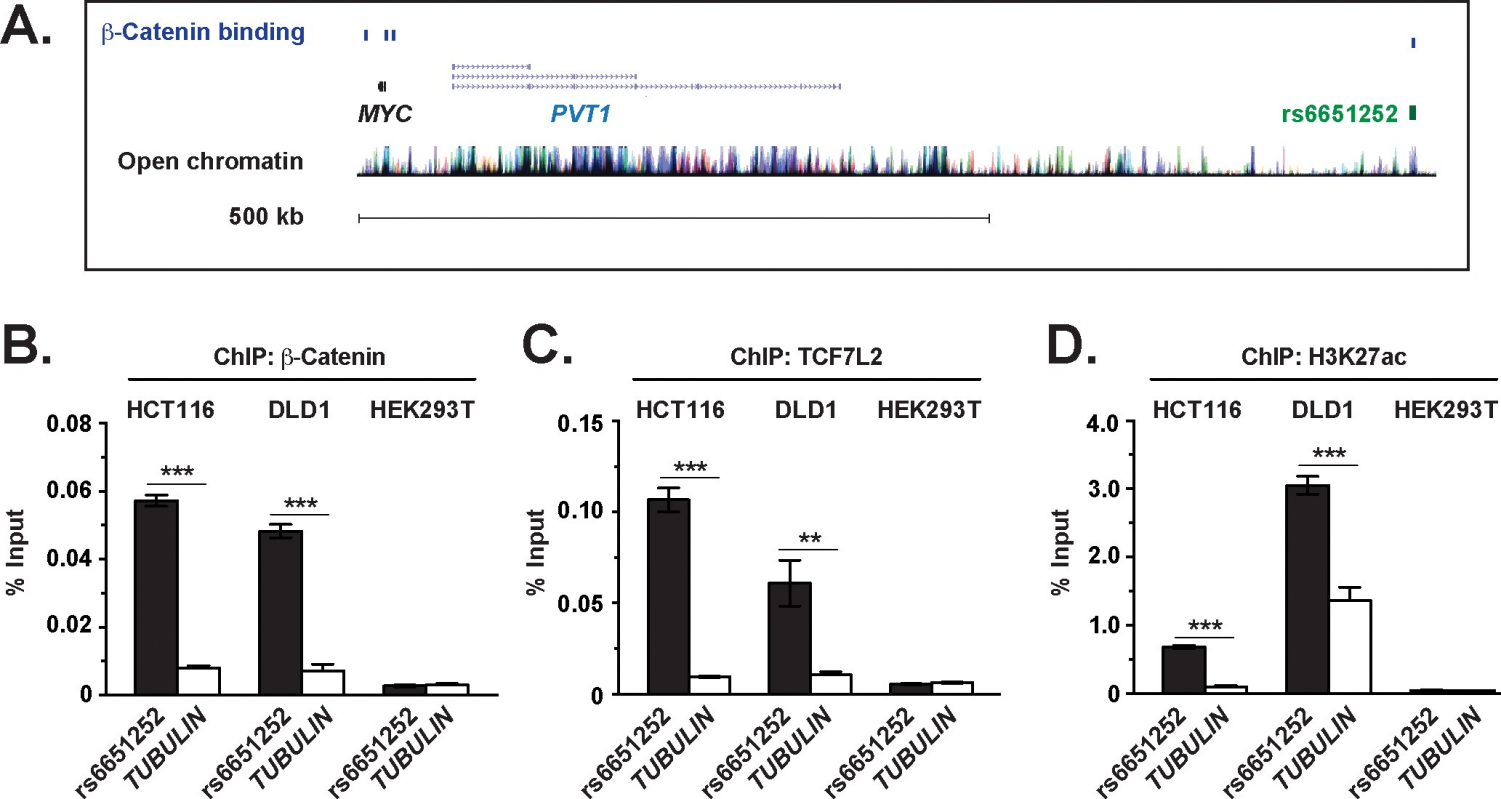
β-Catenin and TCF7L2 bind the rs6651252 locus in colonic epithelial cells. (A). Diagram of the chromosomal *MYC* locus. Shown is the position of rs6651252 relative to *MYC* and *PVT1* genes on chromosome 8. Blue rectangles above depict positions of ChIP-Seq peak regions for β-catenin in HCT116 cells. Clustered vertical lines at bottom indicate H3K27 acetylated regions of chromatin that were downloaded from the UCSC Genome Browser, build hg18 (http://genome.ucsc.edu/). (B). qPCR analysis of DNA fragments precipitated with α-β-catenin antibodies in ChIP assays conducted in HCT116 and DLD-1 colonic epithelial cell lines, and in HEK293T cells. Oligonucleotides used in the PCR reactions flanked rs6651252 or a region in *TUBULIN* as a negative control. (C) and (D)., as in (B) except α-TCF7L2 or α-H3K27Ac antibodies were used in the ChIP assays, respectively. The data are presented as percent of input with error bars representing SEM (**P <0.01; ***P <0.001).

In our prior work, we conducted a ChIP-Seq screen to identify β-catenin-bound genomic regions in an colonic epithelial cell line [27]. We hypothesized that some of these regions could demarcate enhancer elements that contain IBD-associated SNPs. By overlapping β-catenin ChIP-Seq peak regions with positions of IBD SNPs, we identified the CD-associated SNP, rs6651252, as a leading candidate (Fig 1A). Analysis of ENCODE data deposited on the genome browser (http://genome.ucsc.edu/) found that elevated levels of H3K27 acetylated histones, which are a marker of active enhancers, colocalized to this region [34,35]. To confirm these findings, we conducted ChIP-qPCR assays in the HCT116 and DLD-1 colonic epithelial cell lines and used the non-intestinal cell line, HEK293T, as a control. Using primers that flanked rs6651252, we detected robust β-catenin and TCF7L2 binding to this region in the colonic epithelial cell lines and not HEK293T (Fig 1B and 1C). Very little signal was detected using primers that annealed to a region in the *TUBULIN* gene, which attests to the specificity of our ChIP assays. Furthermore, we detected elevated levels of H3K27 acetylated histones at rs6651252 in the intestinal cell lines and not HEK293T, suggesting that this CD-associated SNP may localize to an active and cell-type specific regulatory DNA enhancer element (Fig 1D) [34,35].

### TCF7L2 binds DNA harboring disease-associated rs6651252 variant with stronger affinity

**Fig 2:**
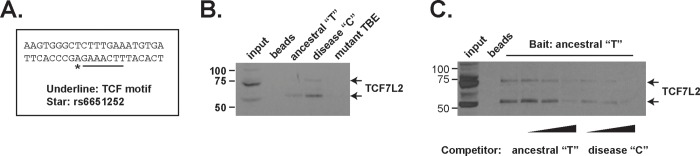
TCF7L2 binds DNA harboring disease-associated rs6651252 variant with stronger affinity. (A). DNA sequence flanking rs6651252 (asterisk) and the adjacent TCF consensus motif (underlined). (B). Western blot analysis of TCF7L2 from HCT116 protein lysates that were incubated with the biotinylated DNA probes indicated and subsequently precipitated with streptavidin conjugated magnetic beads. A probe containing mutations in the consensus TCF binding element (mutant TBE) was used as a negative control. (C). as in (B) except the biotinylated probe harboring the ancestral T variant was used in all reactions. As indicated below, increasing concentrations of unlabeled probes containing T or C were included in the binding reactions prior to precipitation.

The rs6651252 SNP maps immediately adjacent to a consensus TCF binding motif (Fig 2A) [27]. The ancestral allele at this position is a T while the disease-associated allele is a C with a minor allelic frequency of 0.14 [7]. We conducted DNA pull-down assays to determine whether TCF7L2 bound to this fragment of DNA and to test whether the rs6651252 allelic variants impacted its association. Nuclear protein lysates from HCT116 cells were incubated with biotinylated DNA probes, the complexes were precipitated using streptavidin-conjugated magnetic beads and eluted proteins were subjected to western blot analysis. We found that TCF7L2 bound to this element and that mutating the TCF consensus motif blocked its association (Fig 2B). Moreover, the probe containing the C variant precipitated more TCF7L2 compared to the probe containing the T variant (Fig 2B). In addition, we found that adding increasing amounts of unlabeled C probe more effectively competed with TCF7L2 binding to the biotinylated T probe in comparison to reactions containing equivalent amounts of unlabeled T probes (Fig 2C). Therefore, the C variant of rs6651252 potentiates binding of TCF7L2 to this DNA element.

### rs6651252 demarcates a Wnt responsive DNA enhancer element

**Fig 3:**
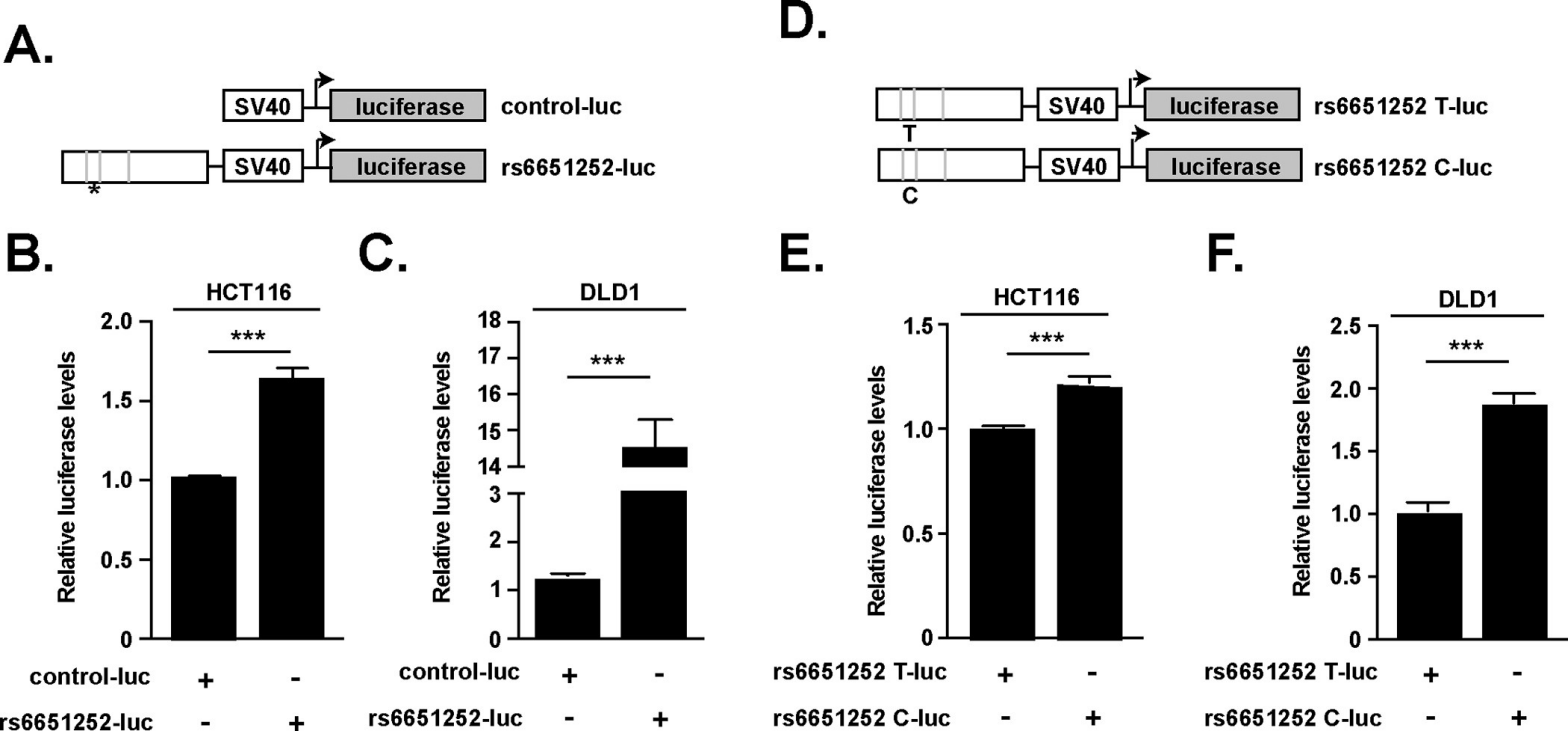
rs6651252 demarcates a Wnt-responsive DNA enhancer element. (A). Diagram of luciferase reporter vectors used in (B) and (C). The pGL3-promoter vector backbone served as a control (control-luc). A 555 bp DNA fragment encompassing rs6651252 was inserted upstream of the SV40 promoter in the pGL3-promoter plasmid vector. Grey vertical lines depict consensus TCF motifs with an asterisk depicting rs6651252. (B) and (C). Luciferase assays conducted in the indicated cell lines with values obtained from rs6651252-luc transfected cells normalized to values obtained from cells transfected with control-luc (D). Diagram of rs6651252 luciferase vectors used in (E) and (F). The T variant of rs6651252 was changed to the C variant using site-directed mutagenesis. (E) and (F). As in (B) and (C), except the indicated cells were transfected with rs6651252 harboring either ancestral T or disease-associated C variant. Error bars are SEM (***P <0.001).

We next used heterologous luciferase reporter assays to determine whether rs6651252 was embedded within a DNA enhancer element. Using genomic DNA isolated from a human colonic epithelial cell line, we PCR amplified a 555 bp segment containing rs6651252 and inserted it upstream of the minimal SV40 promoter in the pGL3-luciferase vector (Fig 3A). We refer to this plasmid as rs6651252-luc and upon sequencing the insert, it contained the T ancestral allele. In comparison to HCT116 and DLD-1 cells transfected with the vector backbone alone, rs6651252-luc drove higher levels of luciferase (Fig 3B and 3C). In HEK293T cells, rs6651252-luc produced lower levels of luciferase relative to the control vector (S1 Fig). Next, we used site-directed mutagenesis to substitute the rs6651252 T variant with the disease-associated C variant (Fig 3D). In both HCT116 and DLD-1 cells, this plasmid drove higher levels of luciferase relative to rs6651252 containing the T variant (Fig 3E and 3F). These results demonstrate that a DNA segment harboring rs6651252 is a cell-type specific enhancer element and that the C variant augments its activity.

### The rs6651252 WRE regulates *MYC* gene expression

**Fig 4:**
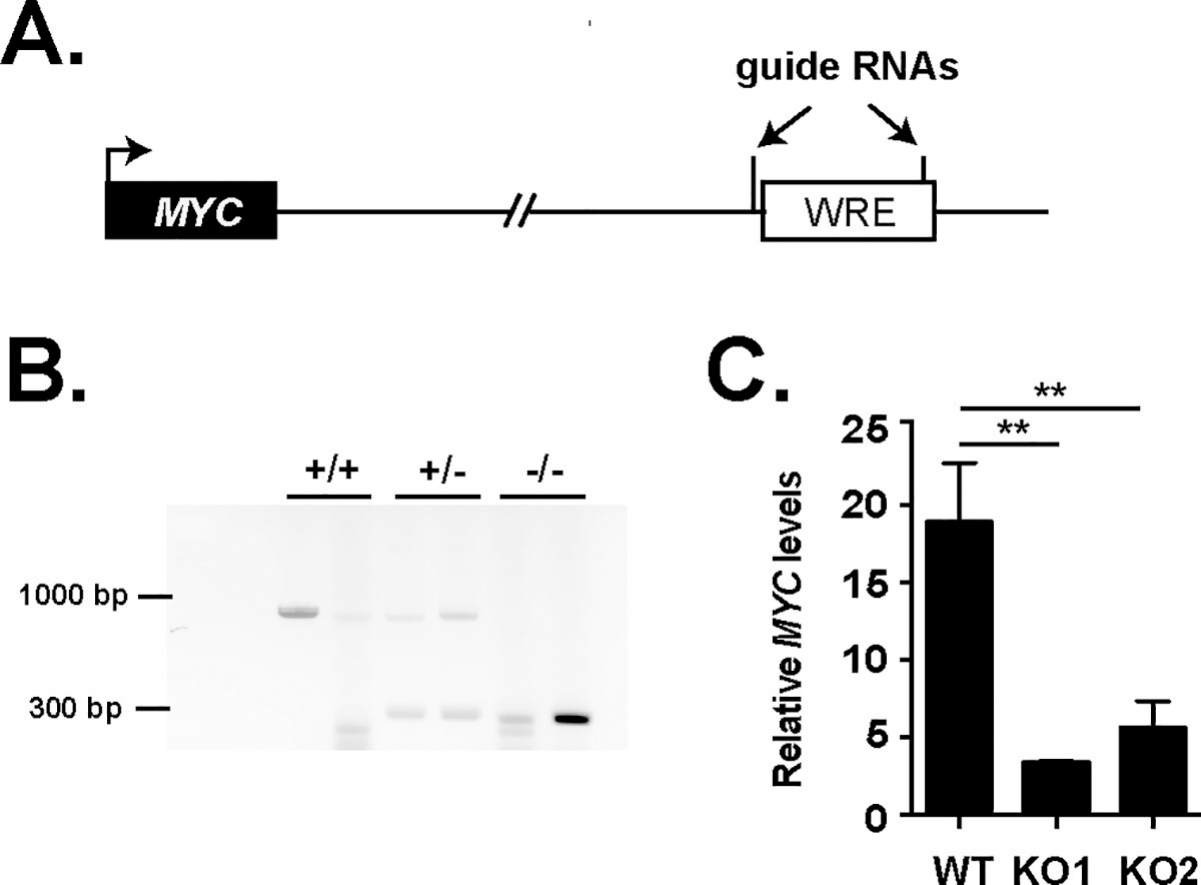
The rs6651252 WRE regulates *MYC* gene expression. (A). Diagram of the *MYC* chromosomal locus with the position of the rs6651252 WRE located approximately 750 kb downstream. The positions of the guide RNAs used for CRISPR/Cas9-mediated deletion are indicated. (B) Agarose gel of PCR products generated from genomic DNAs isolated from clones of HCT116 cells engineered to delete the rs6651252 WRE using CRISPR/Cas9-mediated gene editing. Primers that flanked the rs6651252 WRE were used in PCR reactions. The +/+ indicates clones with non-edited rs6651252 WREs, whereas +/- and -/- indicate heterozygous and homozygous deleted clones, respectively. (C). RT-qPCR analysis of *MYC* mRNA levels in rs6651252 WRE wildtype (WT) and knockout (KO1 and KO2) clones. Values are normalized to average *GAPDH* and *TUBULIN* levels. Error bars denote SEM (**P <0.01).

In a recent study, Meddens et al. used 4C-seq to identify the gene targets of enhancer elements containing embedded IBD-associated SNPs [36]. In that report, rs6651252 was found juxtaposed to the *MYC* and *POU5F1B* gene loci on chromosome 8 through long-range chromatin loops [36]. Whether the rs6651252 enhancer regulated *MYC* or *POU5F1B* expression was not demonstrated. To test if these genes were regulated targets, we used CRISPR/Cas9 to delete the rs6651252 enhancer in the HCT116 cell line (Fig 4A) [29]. After propagating independent clonal lines, we used PCR to assess rs6651252-enhancer status. We successfully identified multiple lines with either heterozygous or homozygous deletions (Fig 4B). The PCR-based genotyping was confirmed using Sanger sequencing. In comparison to a control clone that lacked deletions, two independent knockout clones displayed reduced levels of *MYC* expression as assessed by RT-qPCR (Fig 4C). Deletion of the rs6651252 WRE did not significantly impact *POU5F1B* gene expression levels in HCT116 cells (S2 Fig).

**Fig 5:**
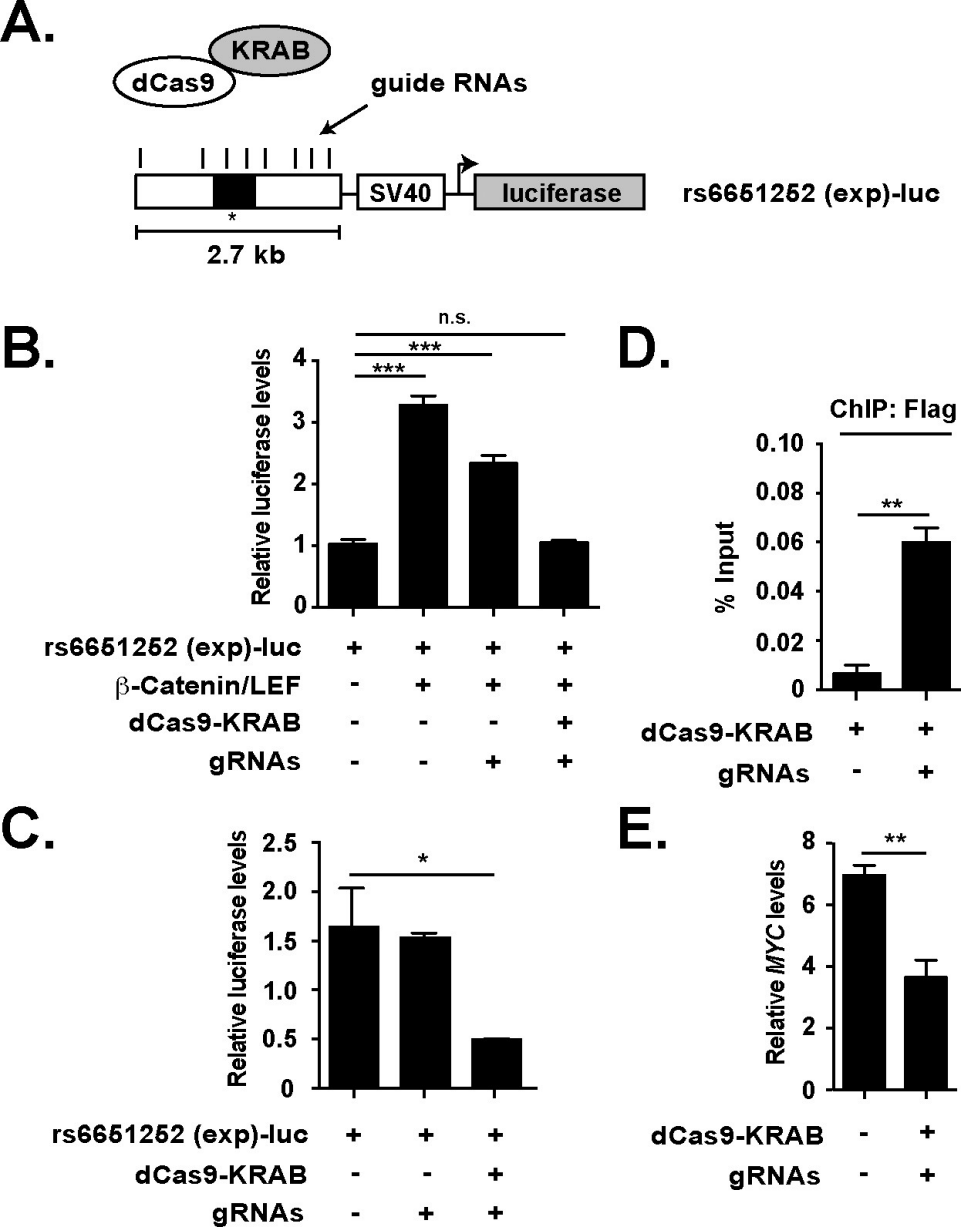
Epigenetic repression of rs6651252 reduces *MYC* gene expression. (A). Diagram of a luciferase reporter vector containing a 2.7-kb DNA fragment encompassing rs6651252. The vertical lines above depict the positions of multiple guide RNAs designed to target the element. These guide RNAs recruit the dCas9-KRAB fusion protein to rs6651252 in transfected cells. (B). Luciferase reporter assays conducted in HEK293T cells. Where indicated, plasmids encoding β-catenin S45F, LEF, dCas9-KRAB and plasmids expressing the guide RNAs (gRNAs) were co-transfected. Data are normalized to luciferase levels in cells receiving the reporter vector alone. (C). Relative luciferase reporter activity in HCT116 cells transfected with the plasmids indicated. (D). qPCR analysis of DNA fragments precipitated with a α-FLAG antibody in ChIP assays conducted in HEK293 cells expressing gRNAs alone or gRNAs and FLAG-dCas9-KRAB. The oligonucleotides used for detection amplified a region encompassing rs6651252. (E). RT-qPCR analysis of *MYC* transcript levels in HCT116 cells expressing gRNAs and the dCas9-KRAB fusion. Non-transfected cells served as a control. Values are normalized to *TUBULIN*. In (B-E), error bars are SEM (*P <0.05, **P <0.01, ***P <0.001).

To confirm that *MYC* gene expression is regulated by the distal rs6651252 WRE, we used a CRISPR/Cas9-based approach to epigenetically repress the function of this DNA regulatory element [37]. In this assay, guide RNAs are used to recruit a mutant Cas9 (dCas9, lacking endonuclease activity) fused to the KRAB transcriptional repressor. We generated eight guide RNAs that tiled a 2.7 kb segment to target dCas9-KRAB to the rs6651252 locus (Fig 5A). Prior to using this system, we performed various control experiments to validate the approach. First, we conducted luciferase assays using the same vector backbone used previously in Fig 3, except that the 2.7 kb segment containing rs6651252 was inserted upstream of the SV40 promoter. In transfected HEK293T cells, plasmids containing β-catenin and LEF (a TCF family member) cDNAs activated expression of luciferase (Fig 5B). Whereas inclusion of plasmids encoding the guide RNAs alone resulted in a slight decrease, co-transfecting guides along with dCas9-KRAB blocked β-catenin/LEF-driven luciferase activity (Fig 5B). Similarly, gRNA/dCas9-KRAB complexes effectively reduced rs6651252 (exp)-driven luciferase levels in HCT116 cells (Fig 5C). Using ChIP-qPCR assays in transfected HEK293T cells, we found that inclusion of the guide RNAs increased levels of dCas9-KRAB recruited to the rs6651252 locus (Fig 5D). Having validated the system, we introduced the guide RNAs and dCas9-KRAB into HCT116 cells and this significantly reduced *MYC* expression in these cells (Fig 5E). Together, these experiments demonstrate that the rs6651252 enhancer regulates *MYC* expression in colonic epithelial cells.

### The rs6651252 C variant correlates with increased *MYC* expression in patient colonic tissues

**Fig 6:**
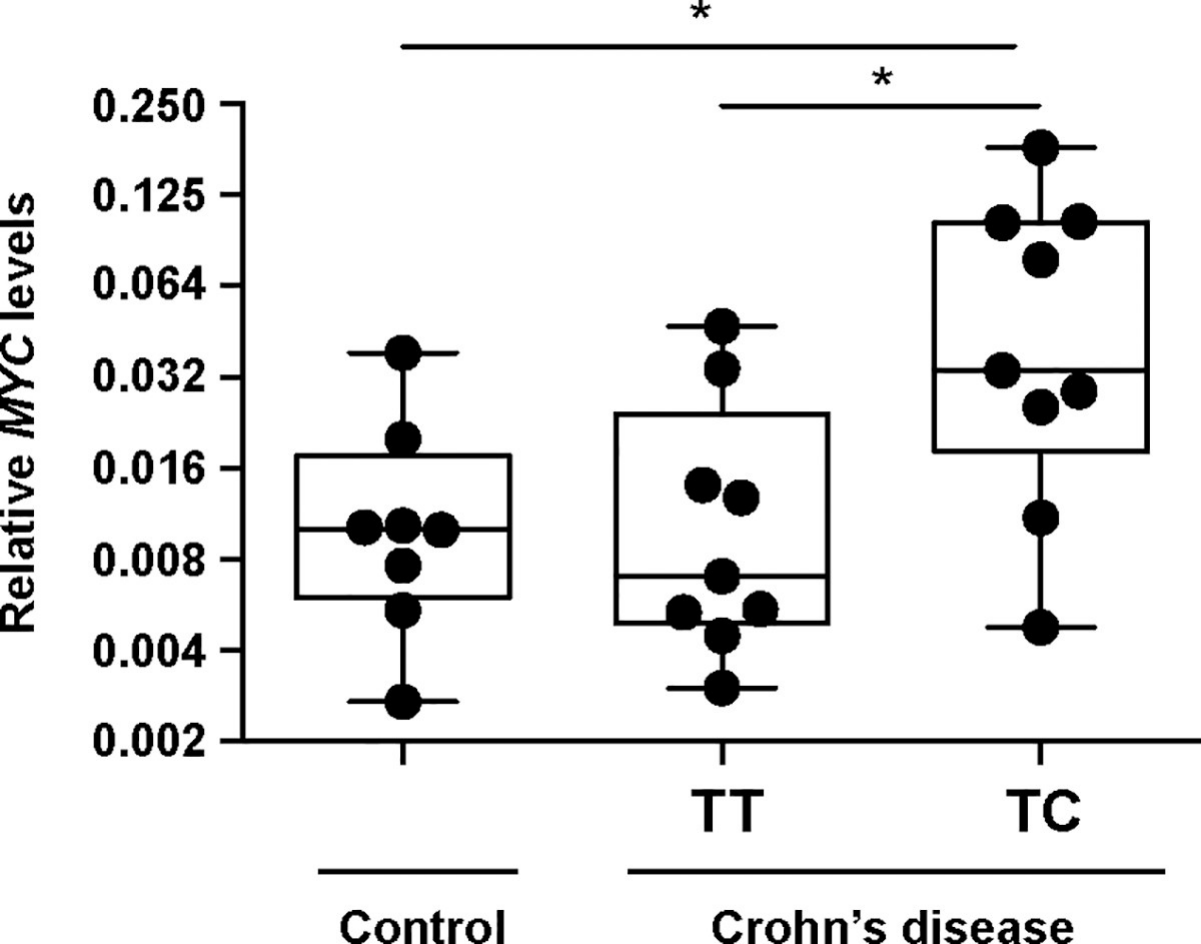
The rs6651252 C variant correlates with elevated *MYC* expression in patient colonic tissues. RT-qPCR analysis of *MYC* transcript levels in resected full-thickness colonic tissues from control and CD patients. For CD patients, the genotype of rs6651252 is indicated. Values are normalized to GAPDH. Error is SEM (*P <0.05).

To determine whether rs6651252 impacts *MYC* gene expression *in vivo*, we obtained colonic segments that were surgically resected from CD patients. As controls, we obtained intestinal tissues from patients that underwent resection for non-IBD related issues such as slow transit or volvulus. We genotyped rs6651252 in genomic DNAs isolated from collected blood and found that while all control patients were homozygous for the ancestral T variant, half of the CD patients were TT and the other half were heterozygous TC. We then isolated RNA from flash-frozen tissues and assessed *MYC* gene expression using RT-qPCR. This analysis found that tissues from CD patients that were heterozygous for the risk variant (TC) contained higher levels of *MYC* transcripts compared to tissues from CD patients harboring homozygous alleles (TT) or controls (Fig 6).

## Discussion

GWAS have identified over 200 SNPs that are associated with a predisposition for developing IBD [3,6–8]. While some of these are found within protein-coding regions of the genome, most map to intergenic and gene-poor loci [3]. Recent work has shown that many of these non-coding SNPs are found within regions of accessible chromatin, demarcated by elevated levels of H3K27ac [13]. Most often, these SNPs are assumed to impact the nearest gene promoter [2,3]. However, it is known that enhancers are capable of impacting more than one gene and can bypass the nearest gene to influence expression of a neighboring gene [38]. Due to the inherent difficulty in studying non-coding regions of DNA, particularly those that may function in a cell-type specific manner, the causative impact of these non-coding SNPs on gene function largely remains a mystery. This current study focused on the CD-associated SNP, rs6651252 that maps to the 8q24 locus. This locus is a large non-coding region of the genome that contains numerous SNPs that have been shown to impact ovarian, prostate and colorectal cancers [39].

We demonstrate that rs6651252 demarcates a WRE and that the disease-associated allele potentiates enhancer activity through higher affinity binding of TCF7L2. In an earlier study, Meddens et al. reported evidence that this rs6651252 enhancer is juxtaposed to the *MYC* promoter, implicating *MYC* as a direct target of this enhancer [36]. Our work confirms and extends this finding as we found that either deletion or epigenetic silencing of this element reduces *MYC* expression in human colonic epithelial cells. Moreover, in our survey of human colonic tissues resected from IBD patients, we find that the disease-associated rs6651252 increases *MYC* expression. Together, these findings are similar to those reported for the cancer-associated SNP rs6983267, which also resides within this gene desert region on chromosome 8 [40–42]. While the rs6983267 disease-associated allele augments TCF binding and enhancer activity, whether it differentially impacts *MYC* gene expression in normal tissues or cancers is still a matter of debate [43].

While much attention has been given to understanding of the role of MYC in colorectal carcinogenesis, much less is known about its role in IBD [44]. Using the dextran sodium sulfate (DSS) model of acute colitis in mice, we reported that slight elevation of MYC (~2.5 fold) promotes restitution of the colonic epithelium [45,46]. Furthermore, we found that lithium treatment, which inhibits glycogen synthase kinase and stabilizes MYC, confers a favorable response to colonic regeneration after acute DSS-induced damage in mice [47]. These findings indicate that short-term MYC stabilization may provide favorable outcomes in IBD by promoting restitution of the epithelial monolayer [45–48]. However, an earlier study reported that levels of *MYC* transcripts are elevated in intestinal tissues isolated from IBD patients in comparison to controls [49]. Furthermore, higher levels of MYC protein was found in inflamed IBD intestinal tissue in comparison to control, non-IBD colonic segments [49]. In addition, MYC expression is elevated, and the *MYC* chromosomal locus is frequently amplified, in colitis-associated cancer (CAC) [50–52]. Therefore, while transient stabilization of MYC is beneficial, long-term and sustained MYC expression is likely detrimental in IBD and CAC.

Our analysis of surgically resected colonic tissues indicates that CD patients harboring the rs6651252 risk allele display elevated levels of *MYC* transcripts relative to levels found in tissues from CD patients that are homozygous for the ancestral allele or controls (Fig 6). Because we have a limited number of control tissues in our Colorectal Disease Biobank, we were unable to identify control colonic tissues that were heterozygous for the risk allele. This precludes interpretations that elevated *MYC* may contribute to disease onset. However, our analysis of genotyped diseased tissues suggests that elevated *MYC* may contribute to disease pathogenesis in CD patients harboring the risk allele. It follows that patients who are homozygous for the disease variant may have a more pronounced phenotype, and as we continue to recruit patients into our biorepository, we hope to identify such patients and will explore this possibility in a future study.

One limitation of our study of human colonic tissues is that full-thickness specimens were analyzed. While we favor a model whereby the rs6651252 disease associated allele elevates *MYC* expression in the colonic epithelium, we cannot dismiss the possibility that other cell types, such as resident lymphocytes, could be contributing to the overall differences we detect. In support of this possibility, Mokry et al. demonstrated that rs6651252 resides in a region of accessible chromatin in specific CD4^+^ sub-cellular populations [13]. Due to the importance of T-cells in IBD pathologies [53], and the role of Wnt signaling in T-cell biology [54], it is possible that the rs6651252 WRE influences *MYC* expression in cells that function in the adaptive cellular immune response. Purification of specific population of cells from genotyped samples and assessing *MYC* gene expression in these cells is required to fully understand how rs6651252 is impacting CD pathogenesis.

In summary, our work shows that rs6651252 demarcates a WRE within the 8q24 locus. The CD-associated allele facilitates stronger TCF7L2 binding to the WRE, potentiates enhancer activity, and increases *MYC* gene expression. While additional work is needed to further define the cell types in which this rs6651252 WRE operates, and the constellation of genes whose expression it impacts, these findings suggest that CD patients harboring this allele may benefit from therapies that target MYC or MYC-regulated genes. Along these lines, *MYC* gene expression is sensitive to inhibitors that target bromodomain and extra-terminal family of proteins (iBETs), such as JQ1 [55,56]. Our findings presented here support the idea that JQ1 should be further evaluated in pre-clinical mouse models of IBD with the hope that someday it could used to augment current treatment strategies for IBD patients.

## Supporting information

S1 Fig The rs6651252 element is repressive in HEK293T cells. Luciferase assays conduced in HEK293T cells transfected with control-luc or rs6651252-luc plasmids as indicated. Error bars depict SEM (**P < 0.01).
(TIF)

S2 Fig Deletion of the rs6651252 WRE does not affect *POU5F1B* gene expression. RT-qPCR analysis of *POU5F1B* gene expression in parental (wild-type, WT) and rs6651252 WRE knockout (KO) HCT116 cell lines. *POU5F1B* levels are normalized to *GAPDH*. Error bars depict SEM.
(TIF)

S1 Table List of oligonucleotide sequences. (XLSX)

## References

[1] Kaser A, Zeissig S, Blumberg RS (2010) Inflammatory bowel disease. Annu Rev Immunol 28: 573–621. 10.1146/annurev-immunol-030409-101225 20192811 PMC4620040

[2] Khor B, Gardet A, Xavier RJ (2011) Genetics and pathogenesis of inflammatory bowel disease. Nature 474: 307–317. 10.1038/nature10209 21677747 PMC3204665

[3] McGovern DP, Kugathasan S, Cho JH (2015) Genetics of Inflammatory Bowel Diseases. Gastroenterology 149: 1163–1176 e1162. 10.1053/j.gastro.2015.08.001 26255561 PMC4915781

[4] Kaplan GG, Ng SC (2017) Understanding and Preventing the Global Increase of Inflammatory Bowel Disease. Gastroenterology 152: 313–321 e312. 10.1053/j.gastro.2016.10.020 27793607

[5] Ek WE, D'Amato M, Halfvarson J (2014) The history of genetics in inflammatory bowel disease. Ann Gastroenterol 27: 294–303. 25331623 PMC4188925

[6] Jostins L, Ripke S, Weersma RK, Duerr RH, McGovern DP, Hui KY, et al (2012) Host-microbe interactions have shaped the genetic architecture of inflammatory bowel disease. Nature 491: 119–124. 10.1038/nature11582 23128233 PMC3491803

[7] Franke A, McGovern DP, Barrett JC, Wang K, Radford-Smith GL, Ahmad T, et al (2010) Genome-wide meta-analysis increases to 71 the number of confirmed Crohn's disease susceptibility loci. Nat Genet 42: 1118–1125. 10.1038/ng.717 21102463 PMC3299551

[8] Liu JZ, van Sommeren S, Huang H, Ng SC, Alberts R, Takahashi A, et al (2015) Association analyses identify 38 susceptibility loci for inflammatory bowel disease and highlight shared genetic risk across populations. Nat Genet 47: 979–986. 10.1038/ng.3359 26192919 PMC4881818

[9] Consortium EP (2012) An integrated encyclopedia of DNA elements in the human genome. Nature 489: 57–74. 10.1038/nature11247 22955616 PMC3439153

[10] Maurano MT, Humbert R, Rynes E, Thurman RE, Haugen E, Wang H, et al (2012) Systematic localization of common disease-associated variation in regulatory DNA. Science 337: 1190–1195. 10.1126/science.1222794 22955828 PMC3771521

[11] Momozawa Y, Dmitrieva J, Theatre E, Deffontaine V, Rahmouni S, Charloteaux B, et al (2018) IBD risk loci are enriched in multigenic regulatory modules encompassing putative causative genes. Nat Commun 9: 2427 10.1038/s41467-018-04365-8 29930244 PMC6013502

[12] Mokry M, Harakalova M, Asselbergs FW, de Bakker PI, Nieuwenhuis EE (2016) Extensive Association of Common Disease Variants with Regulatory Sequence. PLoS One 11: e0165893 10.1371/journal.pone.0165893 27875544 PMC5119736

[13] Mokry M, Middendorp S, Wiegerinck CL, Witte M, Teunissen H, Meddens CA, et al (2014) Many inflammatory bowel disease risk loci include regions that regulate gene expression in immune cells and the intestinal epithelium. Gastroenterology 146: 1040–1047. 10.1053/j.gastro.2013.12.003 24333384

[14] Barker N (2014) Adult intestinal stem cells: critical drivers of epithelial homeostasis and regeneration. Nat Rev Mol Cell Biol 15: 19–33. 10.1038/nrm3721 24326621

[15] Chia LA, Kuo CJ (2010) The intestinal stem cell. Prog Mol Biol Transl Sci 96: 157–173. 10.1016/B978-0-12-381280-3.00007-5 21075344 PMC4165858

[16] Nusse R, Clevers H (2017) Wnt/beta-Catenin Signaling, Disease, and Emerging Therapeutic Modalities. Cell 169: 985–999. 10.1016/j.cell.2017.05.016 28575679

[17] MacDonald BT, Tamai K, He X (2009) Wnt/beta-catenin signaling: components, mechanisms, and diseases. Dev Cell 17: 9–26. 10.1016/j.devcel.2009.06.016 19619488 PMC2861485

[18] Mosimann C, Hausmann G, Basler K (2009) Beta-catenin hits chromatin: regulation of Wnt target gene activation. Nat Rev Mol Cell Biol 10: 276–286. 10.1038/nrm2654 19305417

[19] Korinek V, Barker N, Moerer P, van Donselaar E, Huls G, Peters PJ, et al (1998) Depletion of epithelial stem-cell compartments in the small intestine of mice lacking Tcf-4. Nat Genet 19: 379–383. 10.1038/1270 9697701

[20] Korinek V, Barker N, Morin PJ, van Wichen D, de Weger R, Kinzler KW, et al (1997) Constitutive transcriptional activation by a beta-catenin-Tcf complex in APC-/- colon carcinoma. Science 275: 1784–1787. 9065401 10.1126/science.275.5307.1784

[21] He TC, Sparks AB, Rago C, Hermeking H, Zawel L, da Costa LT et al (1998) Identification of c-MYC as a target of the APC pathway. Science 281: 1509–1512. 9727977 10.1126/science.281.5382.1509

[22] Yochum GS, Cleland R, Goodman RH (2008) A genome-wide screen for beta-catenin binding sites identifies a downstream enhancer element that controls c-Myc gene expression. Mol Cell Biol 28: 7368–7379. 10.1128/MCB.00744-08 18852287 PMC2593444

[23] Yochum GS, McWeeney S, Rajaraman V, Cleland R, Peters S, Goodman RH (2007) Serial analysis of chromatin occupancy identifies beta-catenin target genes in colorectal carcinoma cells. Proc Natl Acad Sci U S A 104: 3324–3329. 10.1073/pnas.0611576104 17360646 PMC1805576

[24] Dang CV, O'Donnell KA, Zeller KI, Nguyen T, Osthus RC, Li F (2006) The c-Myc target gene network. Semin Cancer Biol 16: 253–264. 10.1016/j.semcancer.2006.07.014 16904903

[25] Eilers M, Eisenman RN (2008) Myc's broad reach. Genes Dev 22: 2755–2766. 10.1101/gad.1712408 18923074 PMC2751281

[26] Rennoll S, Yochum G (2015) Regulation of MYC gene expression by aberrant Wnt/beta-catenin signaling in colorectal cancer. World J Biol Chem 6: 290–300. 10.4331/wjbc.v6.i4.290 26629312 PMC4657124

[27] Bottomly D, Kyler SL, McWeeney SK, Yochum GS (2010) Identification of {beta}-catenin binding regions in colon cancer cells using ChIP-Seq. Nucleic Acids Res 38: 5735–5745. 10.1093/nar/gkq363 20460455 PMC2943592

[28] Yochum GS, Cleland R, McWeeney S, Goodman RH (2007) An antisense transcript induced by Wnt/beta-catenin signaling decreases E2F4. J Biol Chem 282: 871–878. 10.1074/jbc.M609391200 17121828

[29] Rennoll SA, Eshelman MA, Raup-Konsavage WM, Kawasawa YI, Yochum GS (2016) The MYC 3' Wnt-Responsive Element Drives Oncogenic MYC Expression in Human Colorectal Cancer Cells. Cancers (Basel) 8. 10.3390/cancers8050052 PMC4880869 27223305

[30] Konsavage WM Jr., Kyler SL, Rennoll SA, Jin G, Yochum GS (2012) Wnt/beta-catenin signaling regulates Yes-associated protein (YAP) gene expression in colorectal carcinoma cells. J Biol Chem 287: 11730–11739. 10.1074/jbc.M111.327767 22337891 PMC3320921

[31] Yochum GS (2011) Multiple Wnt/ss-catenin responsive enhancers align with the MYC promoter through long-range chromatin loops. PLoS One 6: e18966 10.1371/journal.pone.0018966 21533051 PMC3080403

[32] Schieffer KM, Choi CS, Emrich S, Harris L, Deiling S, Karamchandani DM, et al (2017) RNA-seq implicates deregulation of the immune system in the pathogenesis of diverticulitis. Am J Physiol Gastrointest Liver Physiol 313: G277–G284. 10.1152/ajpgi.00136.2017 28619727 PMC6146301

[33] Sehgal R, Berg A, Polinski JI, Hegarty JP, Lin Z, McKenna KJ, et al (2012) Mutations in IRGM are associated with more frequent need for surgery in patients with ileocolonic Crohn's disease. Dis Colon Rectum 55: 115–121. 10.1097/DCR.0b013e31823ccea8 22228152

[34] Creyghton MP, Cheng AW, Welstead GG, Kooistra T, Carey BW, Steine EJ, et al (2010) Histone H3K27ac separates active from poised enhancers and predicts developmental state. Proc Natl Acad Sci U S A 107: 21931–21936. 10.1073/pnas.1016071107 21106759 PMC3003124

[35] Rada-Iglesias A, Bajpai R, Swigut T, Brugmann SA, Flynn RA, Wysocka J (2011) A unique chromatin signature uncovers early developmental enhancers in humans. Nature 470: 279–283. 10.1038/nature09692 21160473 PMC4445674

[36] Meddens CA, Harakalova M, van den Dungen NA, Foroughi Asl H, Hijma HJ, Cuppen EP, et al (2016) Systematic analysis of chromatin interactions at disease associated loci links novel candidate genes to inflammatory bowel disease. Genome Biol 17: 247 10.1186/s13059-016-1100-3 27903283 PMC5131449

[37] Komor AC, Badran AH, Liu DR (2017) CRISPR-Based Technologies for the Manipulation of Eukaryotic Genomes. Cell 169: 559. 10.1016/j.cell.2017.04.005 28431253

[38] Sanyal A, Lajoie BR, Jain G, Dekker J (2012) The long-range interaction landscape of gene promoters. Nature 489: 109–113. 10.1038/nature11279 22955621 PMC3555147

[39] Huppi K, Pitt JJ, Wahlberg BM, Caplen NJ (2012) The 8q24 gene desert: an oasis of non-coding transcriptional activity. Front Genet 3: 69 10.3389/fgene.2012.00069 22558003 PMC3339310

[40] Pomerantz MM, Ahmadiyeh N, Jia L, Herman P, Verzi MP, Doddapaneni H, et al (2009) The 8q24 cancer risk variant rs6983267 shows long-range interaction with MYC in colorectal cancer. Nat Genet 41: 882–884. 10.1038/ng.403 19561607 PMC2763485

[41] Tuupanen S, Turunen M, Lehtonen R, Hallikas O, Vanharanta S, Kivioja T, et al (2009) The common colorectal cancer predisposition SNP rs6983267 at chromosome 8q24 confers potential to enhanced Wnt signaling. Nat Genet 41: 885–890. 10.1038/ng.406 19561604

[42] Wright JB, Brown SJ, Cole MD (2010) Upregulation of c-MYC in cis through a large chromatin loop linked to a cancer risk-associated single-nucleotide polymorphism in colorectal cancer cells. Mol Cell Biol 30: 1411–1420. 10.1128/MCB.01384-09 20065031 PMC2832500

[43] Grisanzio C, Freedman ML (2010) Chromosome 8q24-Associated Cancers and MYC. Genes Cancer 1: 555–559. 10.1177/1947601910381380 21779458 PMC3092220

[44] Sipos F, Firneisz G, Muzes G (2016) Therapeutic aspects of c-MYC signaling in inflammatory and cancerous colonic diseases. World J Gastroenterol 22: 7938–7950. 10.3748/wjg.v22.i35.7938 27672289 PMC5028808

[45] Konsavage WM Jr., Jin G, Yochum GS (2012) The Myc 3' Wnt-responsive element regulates homeostasis and regeneration in the mouse intestinal tract. Mol Cell Biol 32: 3891–3902. 10.1128/MCB.00548-12 22826434 PMC3457533

[46] Konsavage WM Jr., Roper JN, Ishmael FT, Yochum GS (2013) The Myc 3' Wnt responsive element regulates neutrophil recruitment after acute colonic injury in mice. Dig Dis Sci 58: 2858–2867. 10.1007/s10620-013-2686-x 23640071 PMC4104363

[47] Raup-Konsavage WM, Cooper TK, Yochum GS (2016) A Role for MYC in Lithium-Stimulated Repair of the Colonic Epithelium After DSS-Induced Damage in Mice. Dig Dis Sci 61: 410–422. 10.1007/s10620-015-3852-0 26320084

[48] Frey MR (2016) Regenerating Reputations: Are Wnt and Myc the Good Guys After All? Dig Dis Sci 61: 327–329. 10.1007/s10620-015-3947-7 26520110

[49] Ciclitira PJ, Macartney JC, Evan G (1987) Expression of c-myc in non-malignant and pre-malignant gastrointestinal disorders. J Pathol 151: 293–296. 10.1002/path.1711510409 3295155

[50] Robles AI, Traverso G, Zhang M, Roberts NJ, Khan MA, Joseph C, et al (2016) Whole-Exome Sequencing Analyses of Inflammatory Bowel Disease-Associated Colorectal Cancers. Gastroenterology 150: 931–943. 10.1053/j.gastro.2015.12.036 26764183 PMC5270616

[51] Yaeger R, Shah MA, Miller VA, Kelsen JR, Wang K, Heins ZJ, et al (2016) Genomic Alterations Observed in Colitis-Associated Cancers Are Distinct From Those Found in Sporadic Colorectal Cancers and Vary by Type of Inflammatory Bowel Disease. Gastroenterology 151: 278–287 e276. 10.1053/j.gastro.2016.04.001 27063727 PMC5472377

[52] Hartman DJ, Binion DG, Regueiro MD, Miller C, Herbst C, Pai RK. (2018) Distinct Histopathologic and Molecular Alterations in Inflammatory Bowel Disease-Associated Intestinal Adenocarcinoma: c-MYC Amplification is Common and Associated with Mucinous/Signet Ring Cell Differentiation. Inflamm Bowel Dis 24: 1780–1790. 10.1093/ibd/izy057 29788391

[53] Yamada A, Arakaki R, Saito M, Tsunematsu T, Kudo Y, Ishimaru N (2016) Role of regulatory T cell in the pathogenesis of inflammatory bowel disease. World J Gastroenterol 22: 2195–2205. 10.3748/wjg.v22.i7.2195 26900284 PMC4734996

[54] van Loosdregt J, Coffer PJ (2018) The Role of WNT Signaling in Mature T Cells: T Cell Factor Is Coming Home. J Immunol 201: 2193–2200. 10.4049/jimmunol.1800633 30301837

[55] Delmore JE, Issa GC, Lemieux ME, Rahl PB, Shi J, Jacobs HM, et al (2011) BET bromodomain inhibition as a therapeutic strategy to target c-Myc. Cell 146: 904–917. 10.1016/j.cell.2011.08.017 21889194 PMC3187920

[56] Filippakopoulos P, Qi J, Picaud S, Shen Y, Smith WB, Federov O, et al (2010) Selective inhibition of BET bromodomains. Nature 468: 1067–1073. 10.1038/nature09504 20871596 PMC3010259

