# Supplementary figures and images for: The Crohn’s disease associated SNP rs6651252 impacts MYC gene expression in human colonic epithelial cells
Source: PLoS One. 2019 Feb 22;14(2):e0212850. doi: 10.1371/journal.pone.0212850 (PMC6386311; doi:10.1371/journal.pone.0212850)

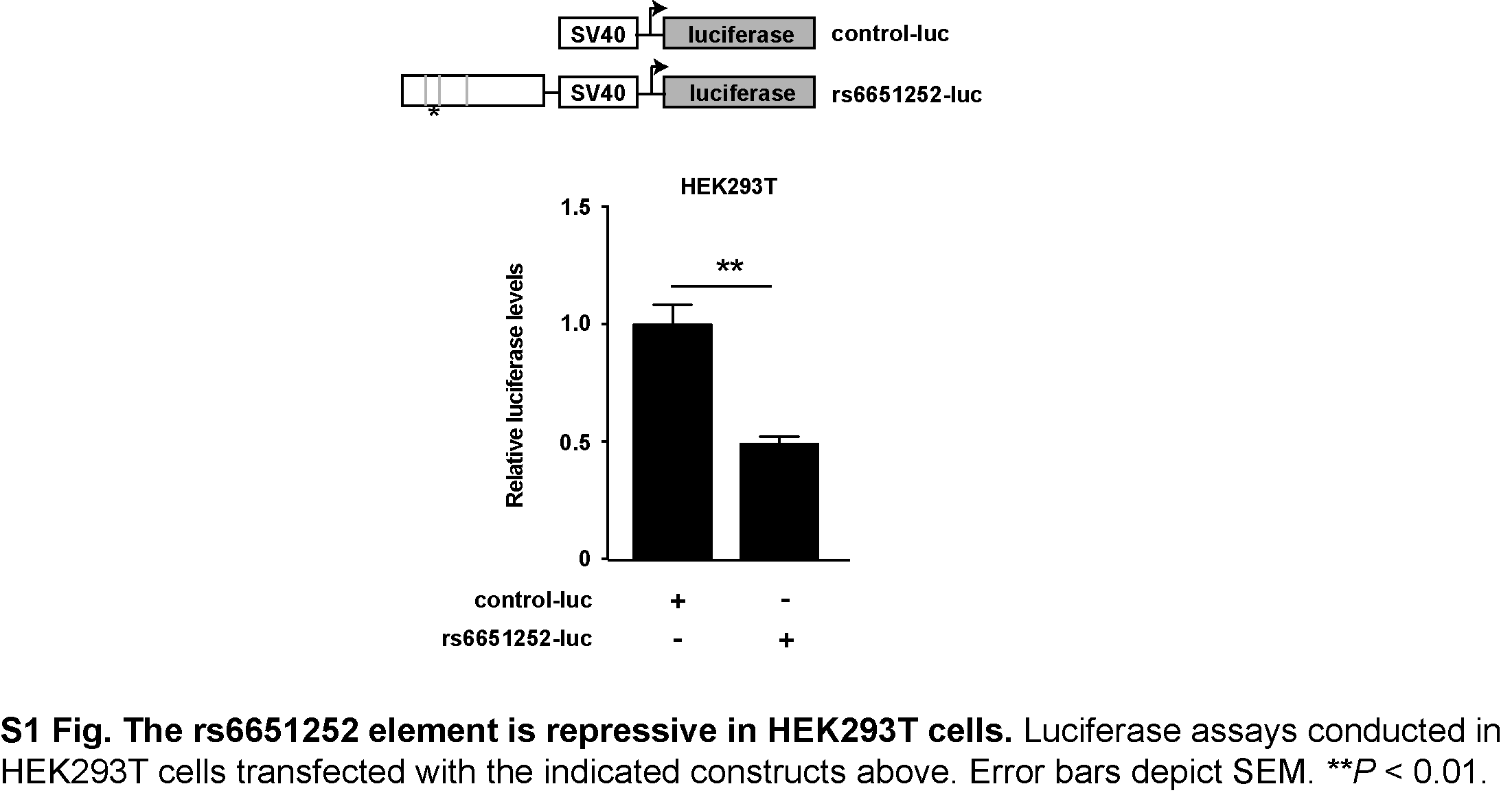

Supplement: S1 Fig — Luciferase assays conduced in HEK293T cells transfected with control-luc or rs6651252-luc plasmids as indicated. Error bars depict SEM (**P < 0.01). (TIF) [file pone.0212850.s001.tif]

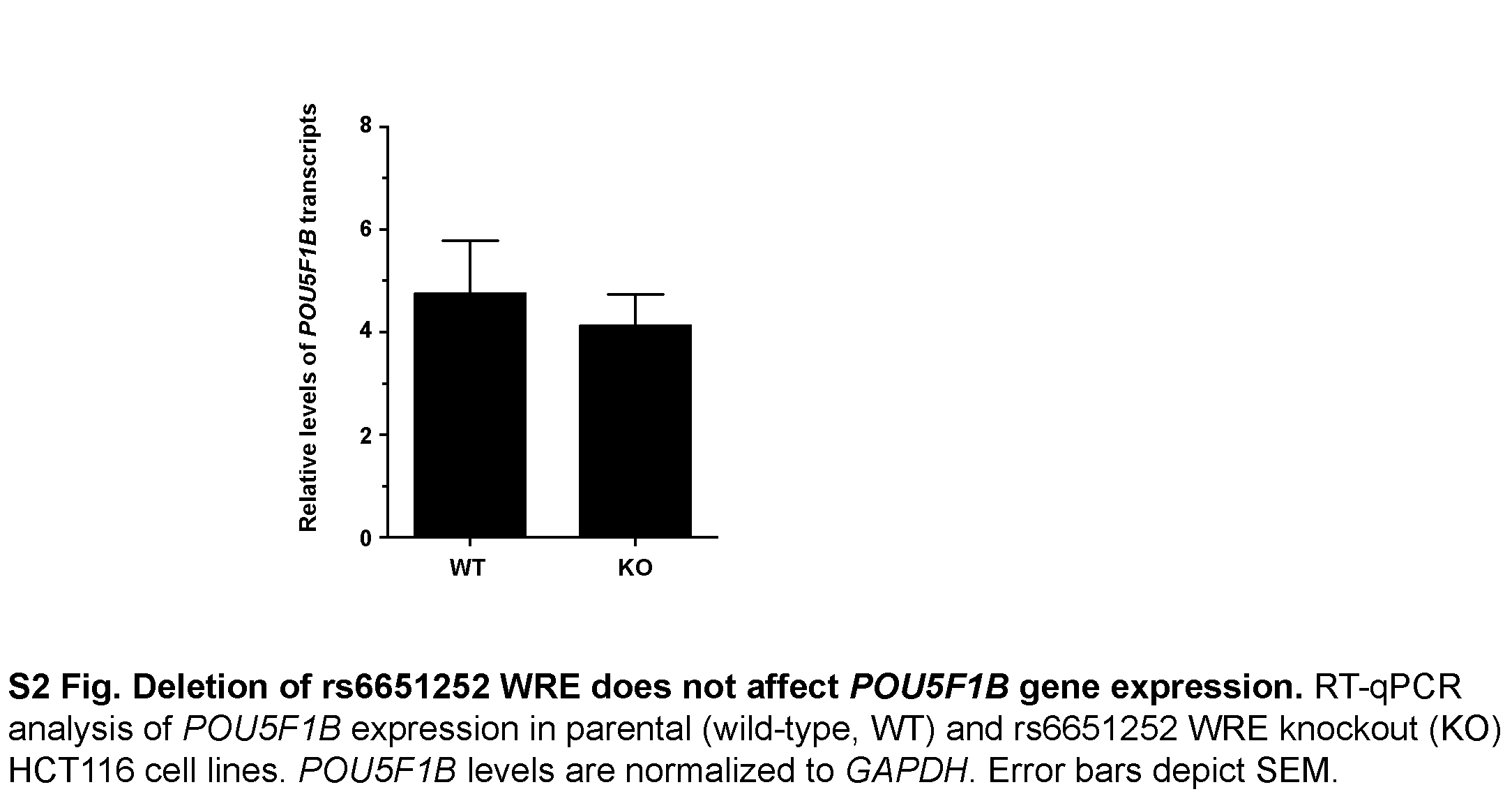

Supplement: S2 Fig — RT-qPCR analysis of POU5F1B gene expression in parental (wild-type, WT) and rs6651252 WRE knockout (KO) HCT116 cell lines. POU5F1B levels are normalized to GAPDH. Error bars depict SEM. (TIF) [file pone.0212850.s002.tif]
